# Boom or burst: integration of CERK1 activation by OXI1 sheds light on ROS signalling during PTI

**DOI:** 10.1093/plcell/koae314

**Published:** 2024-12-04

**Authors:** Rory Osborne

**Affiliations:** Assistant Features Editor, The Plant Cell, American Society of Plant Biologists; School of Biosciences, University of Birmingham, Birmingham B15 2TT, UK

Pattern-triggered immunity (PTI) is activated when conserved, microbe-derived ligands known as pathogen/microbe associated molecular patterns bind to specialized cell-surface receptors called pattern recognition receptors (PRRs). This family of proteins consists of receptor-like kinases and receptor-like proteins whose activation triggers a series of signaling events that lead to reactive oxygen species (ROS) burst, mitogen-activated protein kinase (MAPK) cascades, and the induction of pathogenesis response (PR) genes (DeFalco and Zipfel 2021).

But don’t let the many acronyms deceive you—our understanding of PTI is far from complete. For instance, over 600 receptor-like kinases and 57 receptor-like proteins have been described in the Arabidopsis genome (Hierro et al. 2020). How can their respective activation converge to trigger an almost identical molecular response? **Miaomiao Ma and colleagues (Ma et al. 2024)** sought to understand how plants coordinate complex immune responses, identifying OXIDATIVE SIGNAL-INDUCIBLE 1 (OXI1) as a component of PRR complexes, which integrates pathogen perception from multiple microbes to activate PTI.

Initially curious to identify interactors of CHITIN ELICITOR RECEPTOR KINASE 1 (CERK1), which forms a complex with LYSIN MOTIF RECEPTOR KINASE 5 (LYK5) to recognize fungal chitin, the authors conducted a yeast 2-hybrid screen using the cytoplasmic domain of CERK1 as bait. Among the 18 candidate interactors was OXI1, a serine-threonine kinase previously reported as an essential regulator of oxidative-burst signaling in Arabidopsis (Rentel et al. 2004). After confirming the CERK1-OXI1 interaction in planta (which was notably weaker under chitin treatment), the next step was to investigate if OXI1 contributes to PTI. The authors generated 2 CRISPR mutant lines lacking functional OXI1 (*oxi1-3* and *oxi1-4*) and evaluated their growth, PTI responses, and pathogen susceptibility.

When treated with chitin, both *oxi1* mutants elicited an attenuated immune response, producing a less intense ROS burst, a weaker MAPK cascade, and reduced expression of PR genes compared with wild-type plants. Corroborating their observation that OXI1 interacts with multiple PRRs, mutants were also susceptible to a diverse suite of phytopathogens, suggesting that OXI1 is a positive regulator of PTI that integrates pathogen perception with downstream signaling events.

Further analysis revealed that complementation with native *pOXI1:OXI1* was sufficient to restore the pathogen susceptibility of *oxi1* to that of the wild type, while the kinase-dead *pOXI1:OXI^K45A^* variant could not. As this suggested that the phosphorylation of OXI1 is essential for its function in immunity, the authors next investigated how this kinase might be activated during PTI. Previous reports have shown that OXI1 is phosphorylated in response to H_2_O_2_ (Rentel et al. 2004; Anthony et al. 2006). Given that PTI-induced ROS generation is gated by the NADPH oxidase RbohD, an α-pSer/Thr immunoblot against OXI1-FLAG in *rbohD* protoplasts confirmed the authors' hypothesis that OXI1 phosphorylation is RbohD dependent. Subsequent biochemical analysis also revealed that chitin-induced ROS burst leads to the oxidation of 2 OXI1 cysteine residues, which in turn are critical for its kinase and disease resistance activity.

With OXI1's regulatory role mapped in detail, the final question remained: what are the downstream targets of OXI1 during PTI? Co-immunoprecipitation assays revealed that activated OXI1 directly interacts with MAPKKK5 and RbohD, which it phosphorylates in response to chitin perception. This suggests that OXI1 engages in a positive feedback loop during PTI, as RbohD phosphorylation has been linked to increased ROS generation.

To summarize their findings, the authors describe a model whereby OXI1 transduces the chitin-CERK1 interaction to activate MAPK signaling and ROS generation to support a stronger PTI response (see Figure). Their data also appear to suggest that OXI1 integrates the activation of multiple PRRs by their respective pathogen associated molecular patterns ligands, highlighting it as a critical component of the PTI response. This exciting prospect positions OXI1 as an ideal candidate for targeted breeding programs designed to enhance the resistance of crops to disease.

**Figure.**
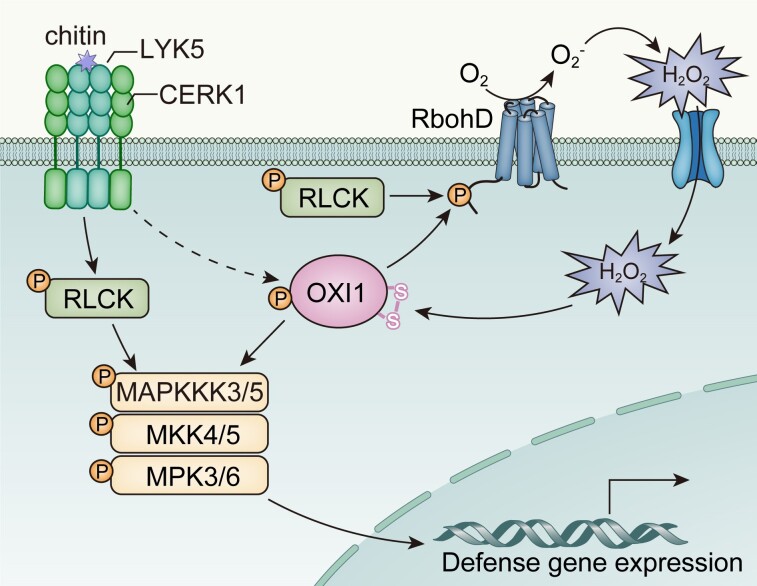
Model proposed by Ma and colleagues describing the role of OXI1 during chitin-mediated activation of PTI via the LYK5/CERK1 receptor complex. Adapted from Ma et al. (2024), Figure 9, copyright American Society of Biologists.

## Dive Curated Terms

The following phenotypic, genotypic, and functional terms are of significance to the work described in this paper:

MAPKKK5 Gramene: AT5G66850
MAPKKK5 Araport: AT5G66850

LYK5 Gramene: AT2G33580
LYK5 Araport: AT2G33580

CERK1 Gramene: AT3G21630
CERK1 Araport: AT3G21630

RBOHD Gramene: At5g47910
RBOHD Araport: At5g47910

OXI1 Gramene: AT3G25250
OXI1 Araport: AT3G25250
